# Rapid Screening for Non-*falciparum* Malaria in Elimination Settings Using Multiplex Antigen and Antibody Detection: Post Hoc Identification of *Plasmodium malariae* in an Infant in Haiti

**DOI:** 10.4269/ajtmh.20-1450

**Published:** 2021-04-05

**Authors:** Lotus L. van den Hoogen, Camelia Herman, Jacquelin Présumé, Ithamare Romilus, Alexandre Existe, Jacques Boncy, Vena Joseph, Gillian Stresman, Kevin K. A. Tetteh, Chris Drakeley, Michelle A. Chang, Jean F. Lemoine, Thomas P. Eisele, Eric Rogier, Ruth A. Ashton

**Affiliations:** 1 Center for Applied Malaria Research and Evaluation, Tropical Medicine Department, Tulane University School of Public Health and Tropical Medicine, New Orleans, Louisiana;; 2 CDC Foundation, Atlanta, Georgia;; 3 Laboratoire National de Santé Publique, Port-au-Prince, Haiti;; 4 Department of Infection Biology, Faculty of Infectious and Tropical Diseases, London School of Hygiene and Tropical Medicine, London, United Kingdom;; 5 Malaria Branch, Division of Parasitic Diseases and Malaria, Centers for Disease Control and Prevention, Atlanta, Georgia;; 6 Ministère de la Santé Publique et de la Population, Port-au-Prince, Haiti

## Abstract

Haiti is targeting malaria elimination by 2025. The Grand’Anse department in southwestern Haiti experiences one-third to half of all nationally reported *Plasmodium falciparum* cases. Although there are historical reports of *Plasmodium vivax* and *Plasmodium malariae*, today, non-*falciparum* infections would remain undetected because of extensive use of *falciparum-*specific histidine-rich protein 2 (HRP2) rapid diagnostic tests (RDT) at health facilities. A recent case–control study was conducted in Grand’Anse to identify risk factors for *P. falciparum* infection using HRP2-based RDTs (*n* = 1,107). Post hoc multiplex *Plasmodium* antigenemia and antibody (IgG) detection by multiplex bead assay revealed one blood sample positive for pan-*Plasmodium* aldolase, negative for *P. falciparum* HRP2, and positive for IgG antibodies to *P. malariae*. Based on this finding, we selected 52 samples with possible *P. malariae* infection using IgG and antigenemia data and confirmed infection status by species-specific PCR. We confirmed one *P. malariae* infection in a 6-month-old infant without travel history. Congenital *P. malariae* could not be excluded. However, our finding—in combination with historical reports of *P. malariae*—warrants further investigation into the presence and possible extent of non-*falciparum* malaria in Haiti. Furthermore, we showed the use of multiplex *Plasmodium* antigen and IgG detection in selecting samples of interest for subsequent PCR analysis, thereby reducing costs as opposed to testing all available samples by PCR. This is of specific use in low-transmission or eliminating settings where infections are rare.

## INTRODUCTION

Although historically considered benign, the severity of non-*falciparum* malaria has now been recognized.^1^ Despite the substantial geographical distribution of *Plasmodium malariae*, global and regional estimates of its prevalence are largely unknown or almost certainly underestimated.^1–5^ *Plasmodium malariae* has been associated with anemia, hospitalization, splenomegaly, kidney damage (specifically in children^6^), and death.^7,8^ There are sporadic reports of congenital *P. malariae* causing fever and anemia in newborn infants.^9,10^

In some cases, *P. malariae* can cause prolonged low-level parasitemia which can remain undetected for years.^11^ These silent infections can threaten malaria elimination efforts in areas where *Plasmodium falciparum* is the dominant species.^12^ Surveillance for non-*falciparum* malaria should therefore not be overlooked in a country attempting to reach malaria elimination. However, affordable methods for identifying these infections in routine surveillance are currently not available. PCR remains impractical at large scale owing to costs, processing time, and the lack of appropriate laboratories in many endemic settings. Multiplex bead assays (MBAs) can be used to rapidly collect antibody and antigen data at scale, thereby selecting samples of interest for confirmation by PCR.^13,14^ Because of the limited incremental costs of adding non-*falciparum* and/or pan-*Plasmodium* targets to a *P. falciparum* antigen and antibody detection panel on an MBA, this is a highly cost-effective approach to identify the presence of possible non-*falciparum* infections.

Today, malaria transmission in Haiti is primarily due to *P. falciparum*, although there are historical reports of *P. malariae* and *Plasmodium vivax*.^15,16^ Evidence for *P. malariae* was found in Haitian refugees arriving in Jamaica in 2004.^17^ Haiti and the Dominican Republic, sharing the island of Hispaniola, aim to eliminate malaria by 2025 (www.malariazeroalliance.org). Although malaria elimination activities target *P. falciparum* and *P. vivax*, the elimination goal includes all *Plasmodium* species. As *P. malariae* has caused outbreaks decades after apparent successful elimination in other regional settings (i.e., Granada and Trinidad^18,19^), it is important to know whether this parasite is endemic. Following the devastating 2010 earthquake in Haiti, rapid diagnostic tests (RDTs) were deployed for malaria diagnosis to supplement microscopy, which was available at some locations in-country. Based on the historical high rates of *P. falciparum*,^16^ RDTs detecting *P. falciparum*–specific histidine-rich protein 2 (HRP2) were deployed; thus, non-*falciparum* infections would be undetected by the primary diagnostic test being used in the majority of Haitian health facilities. Before the earthquake, national malaria prevalence was considered low, though highly focal,^20,21^ but there were concerns about underreporting due to a lack of access to diagnosis and a weak surveillance system.^21–23^

Recent efforts have improved national reporting and access to malaria diagnosis by RDTs. The Grand’Anse department in southwestern Haiti has experienced one-third to half of all the nationally reported *P. falciparum* malaria cases in recent years (source: National Malaria Control Program, Programme National de Contrôle de la Malaria). A case–control study that was conducted as part of operational research efforts to identify risk factors for *P. falciparum* offered an opportunity to test for the presence of non-*falciparum* malaria species.^24^ Post hoc laboratory quantification of HRP2 and pan-*Plasmodium* aldolase (pAldolase) antigens allowed for selection of a subset of samples with possible *P. malariae* infection for species-specific testing of parasite nucleic acids. We aimed to determine the presence or absence of *P. malariae* infection in the selected subset of samples collected in Grand’Anse.

## METHODS

### Study population.

**Figure 1.**
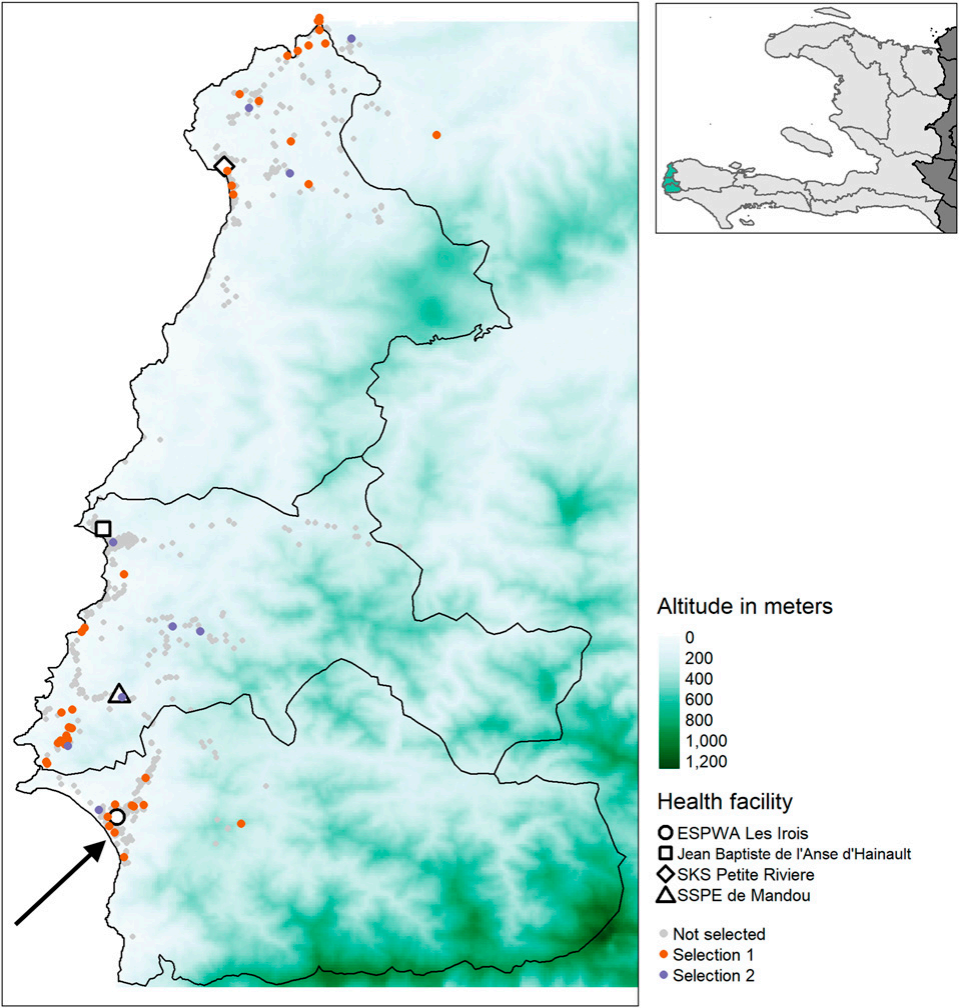
Map of the study area in western Grand’Anse with the included health facilities and household locations of study participants according to the selection criteria for molecular analysis. The inset in the top right shows Haiti (light gray) and neighboring Dominican Republic (dark gray) as well as the study area (green) in the western part of the Grand’Anse department. Participants from the case–control study^24^ were selected for molecular analysis according to the following criteria: Selection 1: pAldolase-positive and PmMSP-1_19_–seropositive participants; Selection 2: PmMSP-1_19_–seropositive and PfMSP-1_19_–seronegative children younger than 15 years. The arrow points at the household location of the 6-month-old infant who tested positive for *Plasmodium malariae* by photo-induced electron transfer PCR (PET-PCR). PmMSP-1_19_: *Plasmodium malariae* 19-kDa fragment of merozoite surface protein 1; pAldolase: pan-*Plasmodium* aldolase; PfMSP-1_19_: *Plasmodium falciparum* 19-kDa fragment of merozoite surface protein 1.

A case–control study was performed in Grand’Anse, southwestern Haiti, as previously described^24^ (Figure 1). In short, individuals attending one of four health facilities between April and July 2018 with suspected malaria (i.e., self-reported history of, or current, febrile illness, assessed by the attending healthcare provider) were invited to participate. The exclusion criteria were younger than 6 months, any severe disease, taking an antimalarial drug in the 14 days before visiting the health facility, and residence outside the commune of the recruiting health facility. Participants with a positive RDT result (SD Bioline Malaria Antigen P.f., 05FK50, Standard Diagnostics; or First Response Malaria HRP2 Antigen detection card test, I13FRC30, Premier Medical Corporation) were selected as cases and RDT-negative participants as controls. Finger-prick blood samples were collected on Whatman 903 cards and stored as dried blood spots (DBSs) at 4°C until processing at the National Laboratory of Public Health (Laboratoire National de Santé Publique, LNSP) in Port-au-Prince.

Basic demographic information, contact details, and approximate home location were collected at the health facility by the study team to enable a follow-up visit to the household. During the follow-up visit, a questionnaire was used to collect information on demographic and behavioral characteristics. All individuals with a positive RDT result received the national first-line malaria treatment during their consultation at the health facility.

### Antigenemia and IgG data collection using a multiplex bead assay.

A MBA was performed to collect HRP2 and pAldolase antigenemia and IgG data to *Plasmodium malariae* 19-kDa fragment of merozoite surface protein 1 (PmMSP-1_19_) and *Plasmodium falciparum* 19-kDa fragment of merozoite surface protein 1 (PfMSP-1_19_) for all study participants (i.e., both cases and controls) using the MAGPIX platform (Millipore Sigma, Burlington, MA). Unique bead regions (MagPlex^®^, Luminex Corp, Austin, TX) were coated by the EDC/Sulfo-NHS intermediate reaction with anti-HRP2 (20 µg per 12.5 × 10^6^ beads mouse IgG anti-PfHRP2 IgG, Abcam) or anti-pAldolase (12.5 µg per 12.5 × 10^6^ beads rabbit IgG anti-pAldolase IgG, Abcam).^13^ Biotinylated detection antibodies (mouse IgG anti-HRP2, Abcam, and rabbit anti-aldolase, Abcam, Cambridge, United Kingdom) were prepared at a final concentration of 1 mg/mL using the EZ-Link Micro Sulfo-NHS-Biotinylation Kit (ThermoFisher Scientific, Waltham, MA). Beads and detection antibodies were transported to LNSP and stored at 4°C.

At LNSP, an MBA was used to collect antigenemia data, as previously described.^25^ Dried blood spots were punched (6 mm discs) and eluted overnight in buffer B (phosphate-buffered saline [PBS] pH 7.2, 0.5% polyvinyl alcohol, Sigma; 0.5% polyvinylpyrrolidine, Sigma; 0.1% casein, ThermoFisher; 0.5% bovine serum albumin [BSA], Sigma; 0.3% Tween 20; 0.1% sodium azide; and 0.1% *E. coli* extract) to a final sample dilution of 1:20 whole blood. Bead mix was prepared in buffer A (PBS pH 7.2, 0.1% Tween 20, 0.5% BSA, and 0.1% sodium azide), beads added to plates (BioPlex Pro, BioRad, Hercules, CA) aiming for 800 beads/region/well, and washed ×2 with 100 µL wash buffer (PBS pH 7.2, 0.15% Tween 20). The samples (50 µL) were incubated with the beads for 90 minutes (all incubation steps were carried out under gentle shaking at ambient temperature) and subsequently washed ×3. Beads were incubated with detection antibodies (50 µL; 1:500 anti-HRP2 and 1:1,000 anti-aldolase in buffer A) for 45 minutes. The plates were washed ×3 and then incubated with streptavidin–phycoerythrin (strep-PE; 1:200 of 1 mg/mL, Invitrogen, Carlsbad, CA) for 30 minutes. The plates were washed ×3 and incubated with a final 30-minute wash step with buffer A. The plates were then washed ×1, resuspended in 100 µL PBS, shaken briefly, and read on a MAGPIX instrument (Millipore Sigma), generating median fluorescence intensity (MFI) with a target of 50 beads/region/well. Median fluorescence intensity values were corrected for responses of a blank well (containing buffer B), providing an MFI-bg signal for analyses. The threshold of positivity was set at the mean +3 SD of the MFI-bg signal of a panel of known negative DBS samples from U.S. residents without history of travel in the last 6 months.^25^ Data were successfully collected for 1,081 participants for PfHRP2 (98%) and 1,059 for pAldolase (96%). Data were excluded for either target if providing a bead count of < 20 within an assay well.

IgG to PmMSP-1_19_^26^ and PfMSP-1_19_^27^ was also collected with the MBA platform, using a previously described protocol^28^ with bead preparation, wash, and incubation methods, as described previously. Proteins were covalently coupled to beads at 20 µg per 12.5 × 10^6^ beads. Dried blood spots were punched (3-mm disc) and eluted in buffer B at 1:100 approximate serum concentration. Bead mixture was prepared, added to plates aiming for 600 beads/region/well, and washed ×2. Sample (50 µL) and anti-IgG (50 µL; 1:500 anti-human IgG, Southern Biotech; 1:625 anti-human IgG_4_, Southern Biotech; 1:200 strep-PE in buffer A) were incubated simultaneously overnight, and the plates were read the next day after washing ×3 and resuspending in PBS. Median fluorescence intensity was corrected for buffer B responses (providing an MFI-bg assay signal) and log10-transformed. A two-Gaussian mixture model of log-transformed data was used to determine the threshold for seropositivity, set at the mean +3 SD of the lower distribution. Sufficient bead counts were available for 1,071 participants for PfMSP-1_19_ and 1,070 for PmMSP-1_19_ (97%).

### *Plasmodium* species specification by photo-induced electron transfer PCR (PET-PCR).

**Figure 2.**
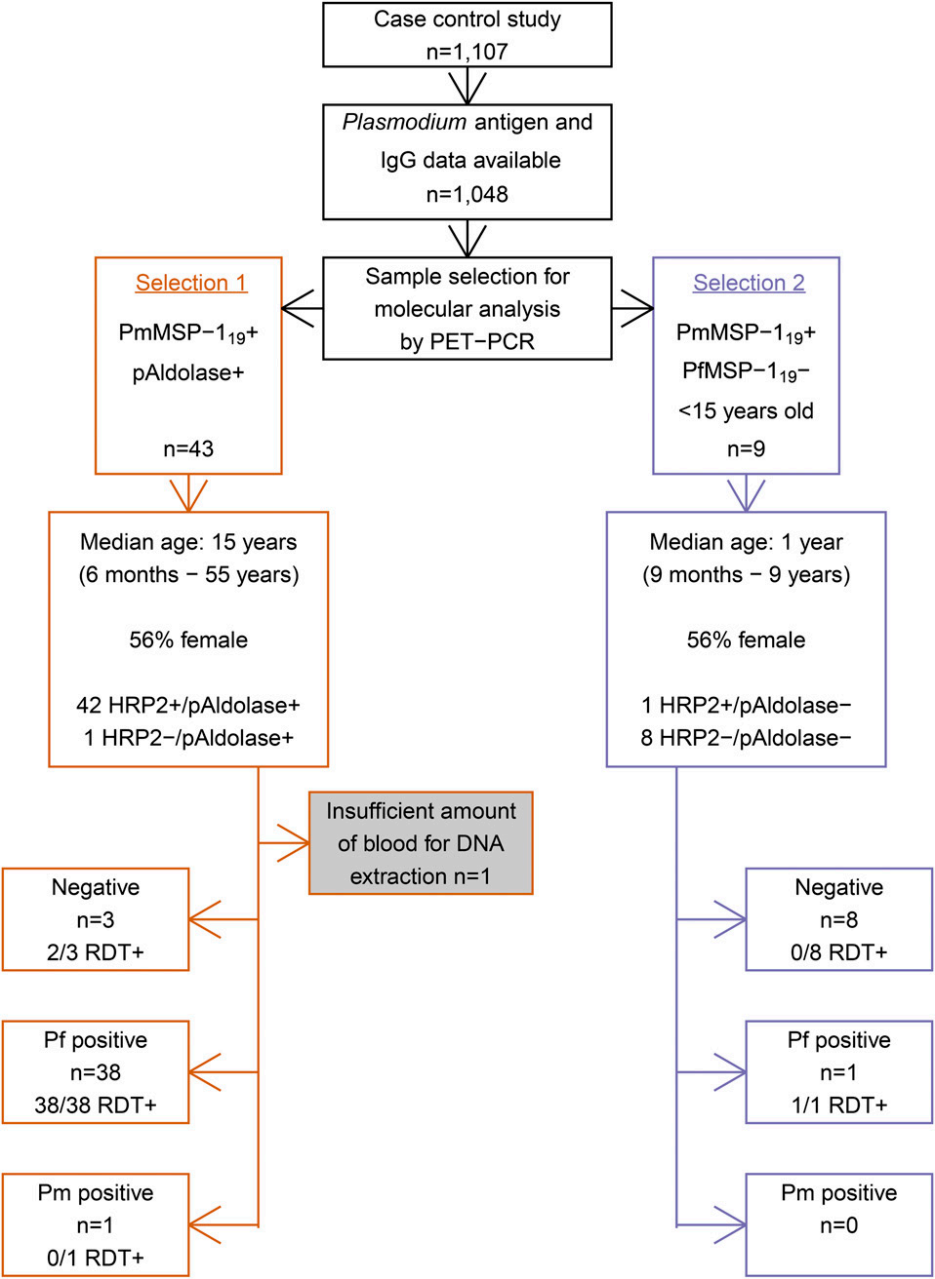
Flowchart of criteria to select study participants for molecular analysis and *Plasmodium* species specification results. Rapid diagnostic tests (RDT; First Response PfHRP2 or SD-Bioline PfHRP2) were performed and dried blood spots (DBSs) collected at the health facility where study participants sought care for current fever or a history of fever. Serological and antigenemia data were measured using the collected DBS at the National Laboratory of Public Health in Port-au-Prince. DBS from selected participants were processed for *Plasmodium* species specification using photo-induced electron transfer PCR (PET-PCR) at the Centers of Disease Control and Prevention in Atlanta, GA. PmMSP-1_19_ = *Plasmodium malariae* 19-kDa fragment of merozoite surface protein 1; pAldolase = pan-*Plasmodium* aldolase; PfMSP-1_19_ = *Plasmodium falciparum* 19-kDa fragment of merozoite surface protein 1; HRP2 = histidine-rich protein 2; *Pf* = *Plasmodium falciparum*; *Pm* = *Plasmodium malariae*; RDT = rapid diagnostic test; + = (sero)positive; - = (sero)negative.

Samples for species specification by PCR were selected according to two sets of criteria using *Plasmodium* antigenemia and IgG results (Figure 2). Dried blood spots from selected participants (*n* = 52) were stored at −20°C until shipment at ambient temperature to the CDC in Atlanta, GA, where they were stored at −20°C until processing.

At the CDC, a PET-PCR assay was performed, as previously described.^29^ DNA was extracted from DBSs using the Qiagen Mini Kit (Qiagen). PCRs for *Plasmodium* genus, *P. falciparum*, and *P. malariae* was performed in 20 μL reactions containing ×2 TaqMan Environmental buffer 2.0 (Applied Biosystems, Foster City, CA). For the genus reaction, 0.5 μL of both forward and reverse (which was labeled with FAM dye) primers at 250 nM were included in each reaction. For the *P. falciparum* primers, 0.5 μL forward primer at 250 nM and 0.25 μL reverse primer at 125 nM (which was labeled with HEX dye) were included. For the *P*. *malariae* primers, 0.5 μL of both forward and reverse (which was labeled with FAM dye) primers at 250 nM were included in each reaction and the sequences were forward (5′-AAGGCAGTAACACCAGCAGTA-3′) and reverse (5′-agg cgc ata gcg cct ggTCCCATGAAGTTATATTCCCGCTC-3′) primers. PCR occurred with the following cycling parameters: initial hot-start at 95°C for 15 minutes, followed by 45 cycles of denaturation at 95°C for 20 seconds, annealing at 63°C for 40 seconds, and final elongation at 72°C for 30 seconds. The cycle threshold (CT) values were recorded at the end of the annealing step. All assays were performed using an Agilent M ×3005pro qPCR system (Agilent Technologies). Positivity of DNA amplification was set at CT < 40.0. The sensitivity and specificity of the PET-PCR assay compared with nested PCR is 92% and 100%, respectively, with an estimated lower limit of detection of two parasites/µL blood.^29^

### Ethical approval.

The study protocol was approved by the National Bioethics Committee of the Haitian Ministry of Public Health and Population (1718-20), Tulane University (2017-366), and the London School of Hygiene and Tropical Medicine (14556). The study protocol and institutional ethical determinations were reviewed and approved by the U.S. CDC, Office of the Associate Director of Science at the Center for Global Health, and CDC investigators were determined not to be engaged in human subject research.

Consent from all participants was recorded electronically on tablets during recruitment at health facilities and confirmed at follow-up interview. Consent for individuals younger than 18 years was given by a parent or guardian. Mature minors (aged 16–17 years and pregnant, a parent, or head of household) were able to provide consent directly.

## RESULTS

### *Plasmodium* antigenemia and IgG results from participants in the case–control study.

**Figure 3.**
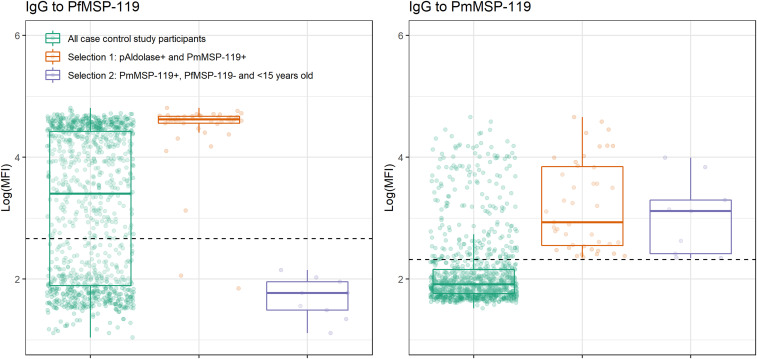
IgG responses to PfMSP-1_19_ and PmMSP-1_19_ in all participants in the case–control study in Grand’Anse, Haiti, and according to the selection criteria for *Plasmodium* species specification using molecular analysis. Box plots and individual IgG results (jittered dots) are shown. Dashed horizontal lines represent the threshold for seropositivity. PmMSP-1_19_ = *Plasmodium malariae* 19-kDa fragment of merozoite surface protein 1; pAldolase = pan-*Plasmodium* aldolase; PfMSP-1_19_ = *Plasmodium falciparum* 19-kDa fragment of merozoite surface protein 1.

Using an MBA, *Plasmodium* antigenemia and IgG data were successfully collected for 1,048 case–control study participants (95%). Data were excluded if insufficient bead counts were available for any of the targets (i.e., < 20 beads/well). In total, 841 participants were negative for HRP2 and pAldolase (HRP2−/pAldolase−); 116 were positive for HRP2 and pAldolase (HRP2+/pAldolase+); 90 were HRP2+/pAldolase−; and one was HRP2−/pAldolase+. In addition, high IgG levels to PmMSP-1_19_ were seen in the study population (Figure 3). Age-specific PmMSP-1_19_ seroprevalence was 0- to 5-year olds: 7% (20/277), 6- to 10-year olds: 9% (15/163), 11- to 20-year olds: 18% (35/195), 21- to 40-year olds: 28% (71/253), and > 40-year olds: 38% (61/160).

### Selection criteria for molecular analysis.

Selection 1 consisted of pAldolase + participants, seropositive to PmMSP-1_19_ (*n* = 43; Figure 2); pAldolase antigenemia would indicate current or recent exposure to *Plasmodium*, whereas PmMSP-1_19_ seropositivity suggests exposure to *P. malariae*. Thus, these might represent single species infection with *P. malariae* (if no HRP2 is detected) but might also be mixed *P. falciparum*/*P. malariae* infections or single *P. falciparum* infections as 98% were HRP2+ (42/43) and 95% were seropositive to PfMSP-1_19_ (41/43; Figure 3).

Selection 2 comprised children ( aged < 15 years) seropositive to PmMSP-1_19_ and seronegative to PfMSP-1_19_ (*n* = 9; Figure 2), removing the possibility of PmMSP-1_19_ seropositivity due to cross-reactivity to PfMSP-1_19_. This selection was focused on children as in adults, seropositivity to PmMSP-1_19_ might indicate a historical infection, whereas in children, it is more likely to be related to a recent or current infection. Of these nine samples, only one was antigen positive: for HRP2. As no further *Plasmodium* antigenemia was detected in this group, the likelihood of detecting a *P. malariae* infection was low but still possible as a chronic low-density *P. malariae* infection, which remained undiagnosed and untreated because of lack of *P. falciparum* exposure (i.e., PfMSP-1_19_ seronegative). Alternatively, these could represent a mixed *P. falciparum*/*P. malariae* infection below the lower limit of detection for pAldolase and/or HRP2 for which seroconversion to PfMSP-1_19_ has not taken place (yet). Lastly, these could represent a single *P. falciparum* infection (for most, below the lower limit of detection of HRP2) for which seroconversion to PfMSP-1_19_ has not taken place (yet).

### Species-specific PCR results in selected participants.

Photo-induced electron transfer PCR confirmed one *P. malariae* infection (Figure 2). The parasite density of this infection was approximately 100 parasites/µL of blood. This was the HRP2−/pAldolase+ sample with PmMSP-1_19_ antibodies that led to the search for *P. malariae* in this population. The *P. malariae* PET-PCR–positive sample was from a 6-month-old boy who was enrolled at the health facility in Les Irois in the southwestern part of Grand’Anse (Figure 1). The infant’s caregiver reported bed net use the previous night. No recent travel was reported for the infant, and no other household members reported fever in the 2 weeks before the interview.

*Plasmodium falciparum* PET-PCR results confirmed HRP2 RDT results, except for two RDT+/PfPCR− samples in the first selection group (Figure 2). Multiplex bead assay results confirmed the presence of HRP2 in these two samples; thus, they could represent recently cleared *P. falciparum* infections with residual HRP2 circulating. An overview of *Plasmodium* serological, antigen and PET-PCR positivity results is shown in Supplemental Table 1.

## DISCUSSION

Here, we report the retrospective detection of a *P. malariae* infection in an infant who participated in a case–control study in Grand’Anse, Haiti, in 2018.^24^ In-country, post hoc *Plasmodium* antigenemia and IgG data collection triggered our search for *P. malariae* in this population.

The confirmed *P. malariae* infection in this study occurred in an infant with a history of fever, seeking care at a local health facility. As the infant tested negative using a *P. falciparum*–specific RDT (i.e., HRP2-based), he would have remained undiagnosed and untreated for malaria. *Plasmodium malariae* infections can lead to hospitalization and have had fatal outcomes even in well-resourced settings.^1^ The fact that no travel history was reported suggests that the infection was locally acquired and thus that *P. malariae* is circulating in this part of Haiti. However, the possibility of congenital malaria could not be excluded as we did not have a sample available from the infant’s mother. It may be possible that the infant’s PmMSP-1_19_–seropositive status was due to maternally derived IgG. Nearly one in five participants in the overall case–control study was seropositive to PmMSP-1_19_. The health facility that the infant attended was located in Les Irois, in the southwestern part of Grand’Anse, which was identified as a focus of *P. falciparum* transmission by Ashton et al.^24^

Data collections in the Artibonite department in 2017 also found evidence of *P. malariae* infections, though rare (M.A. Chang, personal communication). Before this report, the most recent published evidence suggesting the presence of *P. malariae* in Haiti was identification of *P. malariae* infections in Haitian refugees arriving in Jamaica in 2004.^17^ The current surveillance system in Haiti does not support reporting by *Plasmodium* species. Historical reports from Haiti showed that during localized outbreaks in mountainous areas in 1966, about 15% of the malaria infections were due to *P. malariae*, although nationally, they made up 3%.^15^ Overall, in 1964–1965, the monthly incidence of *P. malariae* was found to roughly parallel *P. falciparum* incidence in nationwide surveys in Haiti.^15^

*Plasmodium malariae* parasites are believed to be sensitive to chloroquine treatment, the national first-line malaria treatment in Haiti, although data are limited.^1^ When *P. malariae* presents as a mixed infection with *P. falciparum*, treatment could still occur in Haiti, unless it is a low-density *P. falciparum* infection below the limit of detection of RDTs. However, in the case–control study, 13% of participants seropositive to PmMSP-1_19_ were seronegative to PfMSP-1_19_ (27/202), and therefore, their infections may have escaped detection, and they might not have received antimalarial treatment during their infection. Moreover, *P. malariae* and mixed *P. falciparum*/*P. malariae* infections are frequently of low density and/or asymptomatic; thus, they will likely be missed by RDTs or microscopy.^5^ In the case described here, the infection appears to be a single *P. malariae* infection, though the possibility of a mixed infection with *P. falciparum* below the lower limit of detection of the PET-PCR protocol used (< 2 parasites/µL blood) cannot be excluded. Regardless, the identified *P. malariae* infection was not diagnosed by routine practices at the health facility because of the lack of appropriate diagnostics to detect non-*falciparum* infections.

In other countries in the Caribbean, *P. malariae* has caused outbreaks decades after successful elimination (i.e., Grenada^18^ and Trinidad^19^). These outbreaks were hypothesized to be due to recrudescent infections. *Plasmodium malariae* is known to cause extended periods of latency; one case in Greece had a latency of more than 40 years (and possibly as long as 70 years).^11^ In addition, there is evidence from some settings that *P. malariae* transmission remained or increased while *P. falciparum* decreased.^1,12^ Combined, these reports emphasize the importance of knowing whether *P. malariae* is still endemic in Haiti.

A MBA was used to screen for *Plasmodium* antigenemia and IgG at the Haitian National Laboratory. This enabled us to select relevant samples with possible *P. malariae* infection from the case–control study. As antigenemia data were collected at < 1 United States Dollar per sample^13^ and PmMSP-1_19_ was added to a panel of *P. falciparum* antigens at limited additional costs, this was a highly cost-effective method of selecting possible non-*falciparum* infections for subsequent confirmation by PCR as compared with testing all available samples by PCR.

In addition to the inability to exclude congenital malaria or gather additional information from the *P. malariae* case’s family, a further limitation of our study is the fact that the original study protocol was not designed to determine the presence of non-*falciparum* malaria in this setting. A history of febrile illness was the inclusion criterion for the case–control study, although *P. malariae* is often asymptomatic.^5^ Future efforts to assess the burden of non-*falciparum* malaria in Haiti should therefore (also) focus on asymptomatic populations.

Whether this finding should have policy implications for Haiti is difficult to conclude based on a single identified *P. malariae* case. However, this may have been a locally acquired infection, and there is currently a lack of diagnostic capacity for non-*falciparum* infections in routine care. Considering Haiti’s elimination aims, this preliminary evidence warrants further exploration of the extent of non-*falciparum* transmission, surveillance approaches, and efficacy of first-line treatment against non-*falciparum* species present.

## Supplemental table

Supplemental materials
